# Normative vs personal attitudes toward persons with HIV, and the mediating role of perceived HIV stigma in rural Uganda

**DOI:** 10.7189/jogh.11.04056

**Published:** 2021-09-15

**Authors:** Alexander C Tsai, Bernard Kakuhikire, Jessica M Perkins, Jordan M Downey, Charles Baguma, Emily N Satinsky, Patrick Gumisiriza, Justus Kananura, David R Bangsberg

**Affiliations:** 1 Center for Global Health, Massachusetts General Hospital, Boston, Massachusetts, USA; 2 Mongan Institute, Massachusetts General Hospital, Boston, Massachusetts, USA; 3 Harvard Medical School, Boston, Massachusetts, USA; 4 Mbarara University of Science and Technology, Mbarara, Uganda; 5 Peabody College, Vanderbilt University, Nashville, Tennessee, USA; 6 Last Mile Health, New York, New York, USA; 7 Oregon Health Sciences University-Portland State University School of Public Health, Portland, Oregon, USA

## Abstract

**Background:**

HIV stigma has well-documented negative impacts on HIV testing, transmission risk behavior, initiation of and adherence to antiretroviral therapy, and retention in care. We sought to assess the extent to which anticipated HIV stigma is based on misperceptions of normative attitudes toward persons with HIV, and to determine whether persons with HIV have stronger misperceptions compared with HIV-negative persons or persons of unknown serostatus. We also sought to estimate the association between normative attitudes about persons with HIV and personal attitudes about persons with HIV, and to determine the extent to which anticipated stigma mediates this association.

**Methods:**

We conducted a whole-population survey of 1776 persons living in 8 rural villages in southwestern Uganda. Negative attitudes toward persons with HIV, and anticipated stigma, were measured using a newly validated 15-item scale measuring multiple dimensions of HIV stigma, including social distance, blaming attitudes, and concerns about reciprocity. We used multivariable regression to estimate the association between normative attitudes about persons with HIV and personal attitudes toward persons with HIV, and to determine the extent to which perceptions of normative attitudes (anticipated stigma) mediated this association.

**Results:**

Study participants believed that negative attitudes toward persons with HIV were more pervasive than they actually are. Perceptions of the extent to which these negative attitudes are normative mediated more than one-third of the association between normative attitudes and their personal attitudes. In contrast to what we originally hypothesized, persons with HIV were less likely to misperceive these norms and perceived normative attitudes to be less stigmatizing than did others in the general population.

**Conclusions:**

Interventions designed to accurately describe normative attitudes toward persons with HIV may reduce HIV stigma without directly focusing on the educational components that are typically embedded in anti-stigma interventions.

Of the 29 million persons with HIV in sub-Saharan Africa, only half are aware of their seropositivity, and among these, less than half are on HIV antiretroviral therapy (ART) [1]. Late-stage disease at presentation to care and at ART initiation remain the norm [2]. The stigma attached to HIV has been identified as a critical barrier to improving these HIV prevention and treatment outcomes [3-5]. For example, the ANRS 12249 universal “test and treat” intervention failed to reduce HIV incidence, because fewer than half of those who tested HIV-positive subsequently linked to care [6]. Anecdotal reports suggested that fears of stigma discouraged rapid linkage to care [7]. Treatment refusal despite eligibility has also been documented [8,9]. Meanwhile, HIV remains heavily stigmatized throughout sub-Saharan Africa [10-12]. In some countries such as Uganda, certain stigmas have actually intensified over the past decade [13]. Thus, despite major advances in the use of ART for treatment and prevention, HIV stigma threatens to put the “AIDS-free generation” out of reach.

A critical dimension of the stigma attached to HIV is perceived or anticipated stigma, which occurs when one expects, based on subjective awareness of prevailing social norms, that persons with HIV will be devalued and discriminated against [14]. Numerous studies have examined the health and behavioral impacts of perceived or anticipated stigma [15-22]. In general, these adverse effects are expected to operate whether the stigmatized condition is visible (eg, race/ethnicity) or concealable (eg, asymptomatic HIV) [23,24]. Several studies have also examined the health and behavioral impacts of social norms [25-31], but few studies in the literature on HIV stigma have directly connected the two constructs [29,32,33]. In a well-designed US study of persons with concealable stigmatized identities such as mental illness and family health problems, Quinn and Chaudoir [17] examined how both anticipated stigma and social norms (described in their article as “cultural stigma”) were associated with psychological distress. Hargreaves and colleagues [34] examined HIV-related fears and judgments and perceived stigma, at the community level, in relation to internalized and enacted stigma among people with HIV. They found that internalized and enacted stigma among people with HIV were associated with community-level perceived stigma but not, contrary to what was hypothesized, with community-level fears and judgments. Neither of these studies, however, examined the potentially mediating role of anticipated stigma in explaining the effect of social norms on individual belief. This is an important gap in the literature on HIV stigma because to date the *accuracy* of subjective assessments in the literature on anticipated stigma has not been considered: people may overestimate or underestimate the extent to which others harbor negative attitudes toward persons with HIV or engage in discriminatory behaviors against persons with HIV, and these misperceptions may have important behavioral or psychosocial consequences.

The study in the literature most similar to ours is a study of persons with HIV in which their individual assessments were geographically linked to population-based surveys of randomly selected residents of those same US communities [32,33]. That study–similar in design to Quinn and Chaudoir [17] and Hargreaves and colleagues [34] in its linkage of data between focal study participants (ie, those with the stigmatized condition) and an independent sample of community-dwelling individuals–found that the association between community norms about condom use for HIV prevention and HIV-positive participants’ concern with public attitudes was mediated by HIV-positive participants’ perceptions of the norm [33].

## Hypotheses

We aimed to test the following *a priori* hypotheses, formulated on the basis of prior work. First, misperceptions of normative attitudes and behaviors are common [35-41]. Second, persons with stigmatized conditions more frequently anticipate stigma than they are actually subjected to enacted stigma [42,43]. And third, perceptions of normative attitudes likely mediate the relationship between normative attitudes and personal attitudes [14,44]. Therefore, we hypothesized the following:

H1: People believe negative attitudes toward persons with HIV are more pervasive than they actually are, ie, they misperceive the norm.

H1a: Persons with HIV have stronger misperceptions (about the pervasiveness of negative attitudes toward persons with HIV) compared with persons in the general population who are either HIV-negative or of unknown serostatus.

H2: Normative attitudes about persons with HIV (measured at the community level) are associated with personal attitudes about persons with HIV (measured at the individual level), and the estimated association will be mediated by perceptions of the norm.

Thus, the overall objective of our study was to assess the extent to which perceived HIV stigma in rural Uganda is based on misperceptions of normative attitudes toward persons with HIV, and to determine the extent to which persons with HIV have stronger misperceptions compared with HIV-negative persons or persons of unknown serostatus. We also sought to estimate the association between normative attitudes about HIV and about persons with HIV (measured at the community level) and personal attitudes about HIV and about persons with HIV (measured at the individual level), and to determine the extent to which perceived stigma mediates this association.

## METHODS

### Ethics review

All research assistants received in-depth training on fieldwork and administration of surveys for gathering sensitive information, including instructions on how to temporarily halt the survey if another person came within earshot. They also received two additional training courses from The AIDS Support Organization and a Ugandan counseling psychologist on managing sensitive disclosures by study participants. The survey was framed in general terms as a community survey about the social lives and health of residents of Nyakabare Parish, not as a study about attitudes toward persons with HIV. Each eligible person was approached in the field, typically at their home or place of employment, by a research assistant who spoke the local language (Runyankore). The research assistant described the study in brief and invited their participation. For persons who expressed potential interest, the study was described in detail and their written informed consent to participate was obtained. Study participants who could not write were permitted to indicate consent with a thumbprint.

We solicited feedback on the study design from a community advisory board comprised of eight community leaders (four men and four women), including the district community development officer and an HIV-positive volunteer counselor from a local HIV clinic [45-47]. We also conducted a series of community sensitization meetings in each of the study villages, during which we provided details about the study design, answered questions, and invited feedback [48]. Ethical approval for all study procedures was obtained from the Mbarara University of Science and Technology and the Partners Human Research Committee. Consistent with national guidelines we also obtained clearance to conduct the study from the Uganda National Council of Science and Technology.

### Study population, design, and data collection

Our study was conducted in Mbarara, Uganda, in the southwestern region of the country. The primary commercial hub, Mbarara Town, was listed in the 2014 census as having a population of 195 013 [49], but most residents of the district live in outlying rural villages similar to those in Nyakabare Parish, located approximately 20 km from town. Each of the eight villages is represented in the local council system [50]: Buhingo, Bukuna 1, Bukuna 2, Bushenyi, Nyakabare, Nyamikanja 1, Nyamikanja 2, and Rwembogo. The local economy is largely based on subsistence agriculture, and both food and water insecurity are common [51-53].

The sample size for our study was established by the geographical and political boundaries of the study site. Approximately three months prior to survey administration, we conducted a population census within the parish and enumerated all potentially eligible persons identified in 758 households. Of these, 1942 were considered eligible for the survey: adults aged 18 years and older (or emancipated minors aged 16-18 years) who could provide informed consent and who considered Nyakabare to be their primary place of residence. The remaining persons were determined to be ineligible, typically because they were visitors to the parish but considered their primary place of residence to be outside the parish. We excluded non-emancipated minors; persons who could not communicate with research staff, eg, due to deafness, mutism, or aphasia; and persons with psychosis, neurological damage, acute intoxication, or an intelligence quotient less than 70 (all of which were determined in the field by non-clinical research staff in consultation with a supervisor).

Survey questions were written in English, translated into Runyankore, and then back-translated into English to verify the fidelity of the translation. For translation we employed an iterative process involving in-depth consultation and pilot testing with 18 key informants. The survey was then programmed in the Computer Assisted Survey Information Collection (CASIC) Builder^TM^ software program (West Portal Software Corporation, San Francisco, CA, USA) for data collection in the field by laptop computer.

### Conceptual model

Our analyses were motivated by a conceptual model, derived from published literature, linking prevailing (ie, in the community) negative attitudes toward people with HIV, perceptions of these prevailing attitudes, and individual belief. Drivers of negative attitudes toward persons with HIV vary from setting to setting, but Pryor and colleagues [54] described two broad classes of motivations: (1) instrumental concerns about what it means to interact with a person with HIV, eg, fear of acquisition through casual contact [55,56] and the resulting desire for social distance [57]; and (2) preoccupations with the symbolic meanings of HIV, eg, its association with behaviors perceived to be deviant or immoral [58]. Some drivers are both instrumental and symbolic; for example, in some settings HIV remains symbolically associated with death and premature disability, and related instrumental concerns prompt the exclusion of persons with HIV from local networks of mutual aid [59-61]. The disparate nature of these drivers makes it clear that HIV stigma unfolds in a context within which one group exercises power over another [62]. In general, norms and personal beliefs can be focused either on HIV itself (“HIV is a curse from God”) or about persons with HIV (“persons with HIV are cursed”). For convenience of exposition, in the remainder of this manuscript we refer to normative and personal attitudes in relation to persons with HIV only. When negative attitudes toward persons with HIV manifest in discriminatory behavior – whether through acts of abuse such as threats or violence, or through acts of exclusion such as restricted job opportunities – enacted stigma is said to occur [63]. Internalized stigma, sometimes also described as self-stigma [64], results when persons with HIV accept these negative attitudes as valid and ultimately develop self-defacing beliefs about themselves [44].

The present study focuses on the phenomenon of perceived or anticipated stigma, which occurs when one expects, based on their subjective awareness of prevailing norms, that persons with HIV will be devalued and discriminated against [14]. In a population where persons with HIV are commonly thought to be promiscuous or where persons with HIV are commonly targeted with violent acts, subjective awareness of these normative attitudes and behaviors may lead one to expect that persons with HIV will be rejected and devalued in such an environment. Accordingly, perceived or anticipated stigma is typically measured by assessing whether the survey respondent is subjectively aware of the extent to which others in the population harbor negative attitudes toward persons with HIV, ie, whether the respondent believes such negative attitudes are normative. For example, in the 12-item stereotype subscale by Sayles and colleagues [65], persons with HIV were asked to rate on a five-point Likert scale their agreement with statements such as the following: “People think you can’t be a good parent if you have HIV.” Perceived stigma scale items also frequently imply expectations of devaluation or negative behaviors based on the stigmatized status, such as those illustrated in Genberg and colleagues [66], eg, “Most people would not buy vegetables from a shopkeeper or food seller that they knew had AIDS.”

As these examples of scale items demonstrate, perceived or anticipated stigma can be assessed in diverse samples because subjective awareness of prevailing norms about what constitutes approved or disapproved beliefs or conduct can be measured irrespective of the survey respondent’s serostatus [67-69]. Functionally, these stigma scale items can be understood as eliciting the survey respondent’s perceptions about descriptive or injunctive norms. In the classic typology elaborated by Cialdini and colleagues [70], descriptive norms refer to majority behaviors in a group (or what is commonly *done*: “Most people would not buy vegetables from a shopkeeper or food seller that they knew had AIDS” [66]), while injunctive norms refer to majority attitudes in a group (or what is commonly *believed*: “People think you can’t be a good parent if you have HIV” [65]). For persons with HIV, who have the stigmatized attribute in question, subjective awareness of the norm has a *personalized* and negative valence, and is therefore sometimes also referred to as felt stigma [17,63], felt normative stigma [68], or anticipated stigma. When elicited from persons in the general population (presumed to lack the stigmatized attribute in question), this construct is typically referred to as “perceived stigma” [22,34]. However, whether or not one has the stigmatized status in question, measurement of perceived or anticipated stigma proceeds in the same fashion: by eliciting perceptions of normative attitudes and behaviors. For ease of exposition, in the remainder of this manuscript we will refer primarily to the construct of “perceived stigma” and will reserve the term “anticipated stigma” when discussing this construct from the perspective of persons with HIV.

**Figure 1:**
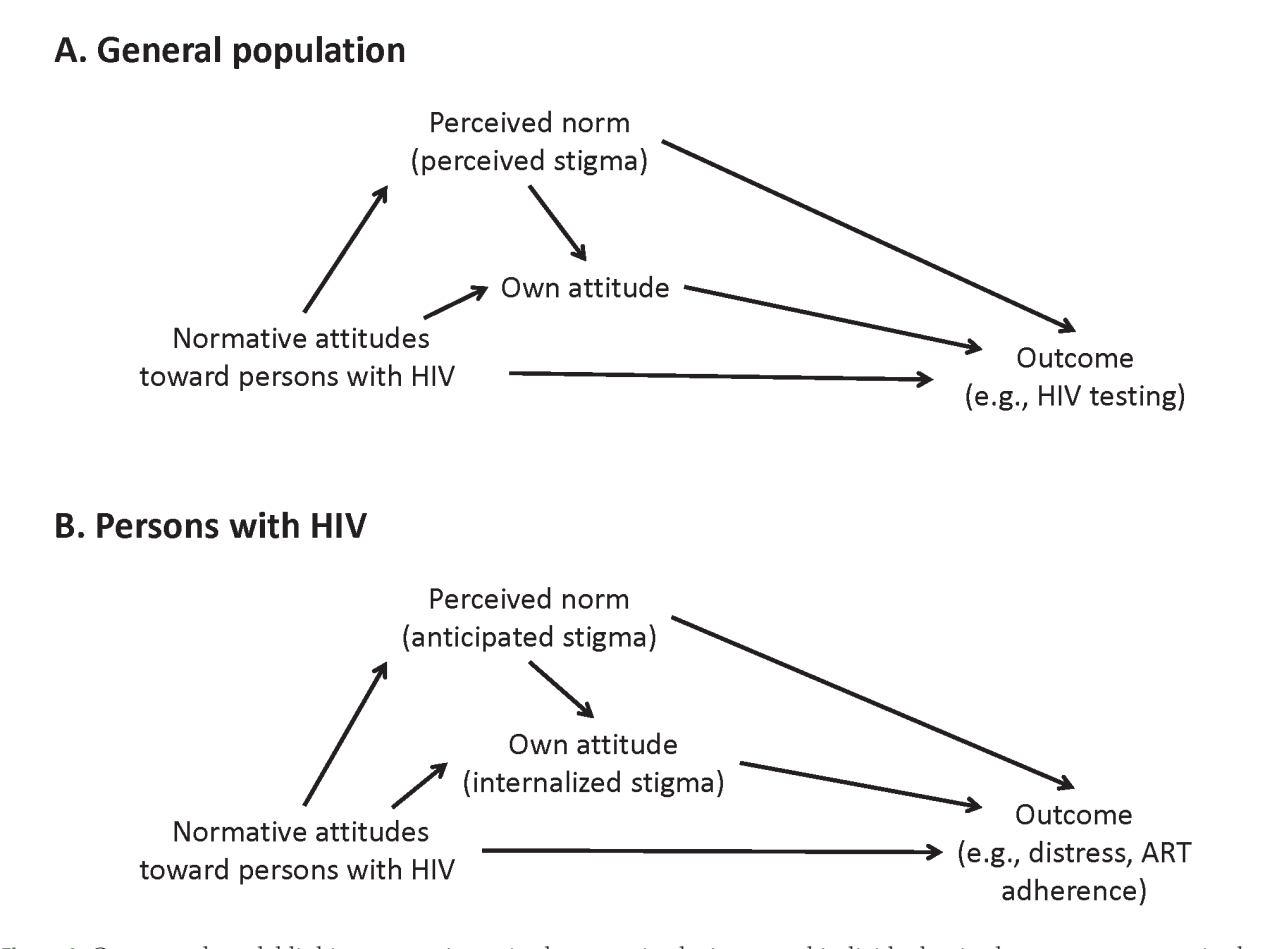
Conceptual model linking normative attitudes, perceived stigma, and individual attitudes, among persons in the general population (**Panel A**) and among persons with HIV (**Panel B**).

Numerous studies have examined how normative attitudes can exert influence on health behavior and health outcomes [25-31]. Perceptions of normative attitudes represent a critical mediator between normative attitudes and HIV-related outcomes of interest (**Figure 1**). Namely, for normative attitudes to meaningfully change individual-level behavioral or psychosocial outcomes, the norms must first be perceived [14,44]. One’s own personal attitudes (or behaviors) may then come to mirror the prevailing norms. The outcome may be contingent on one’s serostatus. HIV-negative persons or persons of unknown serostatus may adopt the same negative attitudes toward persons with HIV. In contrast, persons with HIV may adopt these negative attitudes as valid and develop internalized stigma.

No studies have invoked the concept of perceived norms in trying to understand the behavioral or psychosocial consequences of anticipated HIV stigma. Scambler and Hopkins [63] classically distinguished anticipated (felt) stigma from enacted stigma among persons with epilepsy; they noted that felt stigma was more prevalent than the experiences of being subjected to enacted stigma might suggest. Other studies have empirically demonstrated a similar distinction among persons with mental illness: that persons with mental illness anticipate stigma more frequently than they actually experience enacted stigma [42,43]. Takada and colleagues [71] showed that negative attitudes toward people with HIV are correlated with the beliefs of others within social networks, while Hargreaves and colleagues [34] showed that internalized stigma among people with HIV are correlated with perceived stigma reported by others in their communities. The distinction between normative attitudes vs perceptions of normative attitudes (perceived stigma), in relation to individual belief, is an important one because perceptions about normative attitudes may not accurately reflect the normative attitudes that prevail in the population. For example, while there is evidence that negative attitudes toward persons with HIV have softened over time, there is also evidence that anticipated stigma has actually increased [10,13,72].

Misperceived norms have been most consistently documented in the setting of hazardous alcohol use among young adults in high-income countries [35-37]. Students generally view themselves as drinking less, and being less approving of alcohol use, compared with their peers [38,39]. These misperceptions have important implications for health behavior: students who perceive, contrary to fact, alcohol use as being highly prevalent, and attitudes toward drinking as being highly permissive, are more likely to use alcohol themselves [37,40,41]. Similar findings have been documented with regard to other health risk behaviors (eg, tobacco use [73], heavy alcohol use [74], HIV transmission risk behavior [30,75], perpetration of intimate partner violence [76]) as well as health behaviors (eg, HIV testing [29,77], violence prevention [78]): people who believe that these behaviors are less normative than they actually are may be less likely to engage in these behaviors themselves. Conversely, people who believe that these behaviors are normative are more likely to engage in these behaviors. Derived from this literature is a class of interventions that attempts to reduce health risk behaviors (or enhance health behaviors) by disseminating information about the true prevalence of such behaviors among peers or about the true extent to which peers hold permissive attitudes toward such behaviors [79-81]. A similar class of interventions has been used to curb inappropriate prescribing behavior among clinicians [82]. In the same way, interventions to help people understand their misperceptions about the extent to which others hold negative attitudes toward persons with HIV may provide new opportunities to reduce HIV stigma.

### Primary outcomes

We administered a series of 15 questions to all study participants regardless of serostatus (Table S1 in the **Online Supplementary Document**). These questions were derived from several different sources. Five items on social distancing were derived from Joint United Nations Programme on HIV/AIDS [83] and Steward and colleagues [68]. Five items related to guilt and blame were taken from Genberg and colleagues [66], Kalichman and colleagues [84], and Maughan-Brown [85]. Finally, drawing on a previously published conceptual model of poverty and HIV stigma [59-61], we created five new items related to concerns about the ability of persons with HIV to make reciprocal economic contributions to local networks of mutual aid. These 15 questions were used to measure two constructs, one at the individual level and one at the village level:

1. *Personal attitudes (individual level).* To elicit personal attitudes toward persons with HIV, we asked study participants to indicate whether they agreed or disagreed with each statement (“In your personal opinion, do you agree or disagree with the following statement?”). Persons with HIV who express agreement with these stigmatizing beliefs (eg, “HIV-positive persons are of lower worth compared to HIV-negative persons”) may be conceptualized as having internalized the stigma of HIV [86].

2. *Normative attitudes (village level).* To measure the extent to which these negative attitudes toward persons with HIV were actually normative at the village level, we calculated the proportion of study participants in each village who agreed with each statement.

Next, we administered a parallel set of these 15 items to measure a third construct, at the individual level:

3. *Perceptions of normative attitudes (individual level).* To measure perceptions about whether these attitudes were normative in the village (perceived stigma), we administered a parallel set of these 15 items. However, instead of being asked about their personal beliefs, study participants were instead asked about the beliefs of others: “How many adults who stay in your village, not including yourself, would agree with the following statement?” Responses were scored on a four-point Likert-type scale: “All or almost all; for example, at least 90% of people in my village”; “More than half of people in my village”; “Fewer than half of people in my village”; and “Very few, or no one; for example, less than 10% of people in my village.” Our use of parallel-worded scale items to measure two similar stigma constructs at the individual level–one at the level of individual belief and one at the level of beliefs attributed to others in the community–is most similar to the study by Visser and colleagues [67], in which parallel scales were administered to measure “personal stigma” (negative attitudes toward persons with HIV) and “attributed stigma” (anticipated stigma). In a similar fashion, Steward and colleagues [68] used parallel scales to measure internalized stigma and “felt normative stigma” (anticipated stigma), while Stangl and colleagues [69] used parallel scales to measure internalized, enacted, and perceived stigma.

### Other covariates

The survey instrument contained a number of questions eliciting basic socio-demographic characteristics, including sex, age, educational attainment, and marital status. To measure total wealth, we asked participants a series of 19 questions about household assets and housing characteristics (eg, number of plots of land owned, whether a household member owns a radio, whether their home has a cement floor, etc.). Then, following the method proposed by Filmer and Pritchett [87], we applied principal components analysis to these variables. The first principal component was retained and used to define the wealth index [88]. The wealth index was specified as a continuous variable, with higher values indicating greater asset wealth. Finally, we asked study participants to report their HIV serostatus, with the following response options: HIV-positive, HIV-negative, unknown, or refused. Given that it is rare for persons to identify as HIV-positive when they are actually seronegative [89], study participants were categorized as having “unknown” serostatus if they indicated on the survey that they were HIV negative, did not know their serostatus, or refused to answer.

### Statistical analysis

All analyses were conducted with Stata software (version 14, StataCorp LP, College Station, TX, USA). First we calculated the proportion of study participants who indicated agreement with each of the 15 items. Then we assessed the extent to which study participants perceived these negative attitudes to be normative. Their perceptions of the normative attitude were then compared against the actual prevalence of these attitudes (measured at the village level) to determine the extent of their misperceptions. For example, if a study participant lived in a village where only five percent of study participants agreed that “HIV-positive persons are of lower worth compared to HIV-negative persons” but that study participant selected “more than half” when asked to estimate how many other persons in the village would agree with that statement, then s/he was categorized as having misperceived the norm (ie, because the degree of perceived stigma exceeded the actual village-level prevalence of negative attitudes toward persons with HIV). Analyses were stratified by self-reported HIV serostatus so that we could assess the extent to which misperceptions were stronger among persons with HIV compared with the rest of the community.

We first investigated the factor structure and reliability of the 15-item scale measuring negative attitudes toward persons with HIV. We performed exploratory factor analysis on the scale items, using principal-factors extraction and oblique promax rotation. Because there are no sufficient criteria that can be used in isolation to determine the number of candidate factors for retention, we used three criteria. First, we examined the pattern of factor eigenvalues [90,91]. Second, we graphed the eigenvalues in decreasing order to visually inspect the scree [92]. Third, we examined the loadings of the individual items on the different factors [93]. Cronbach’s alpha was used to assess the internal consistency of the identified factors. We examined item-test correlations, and then re-calculated the Cronbach’s alpha after sequentially deleting each of the items in turn.

After establishing the presence of a single, dominant factor (described in the results below), we generated omnibus measures (across all 15 items) of the intensity of negative attitudes toward persons with HIV, normative attitudes, and perceptions of normative attitudes by following Kling [94] in defining a summary index for each of the three constructs as the equally weighted average of the *z*-scores of its 15 components. While the actual values of the indices carry no meaning, their signs are oriented so that higher values denote greater intensity of negative attitudes toward persons with HIV or perceptions of more stigmatizing norms. Thus, instead of conducting 45 different χ^2^ tests to compare HIV-positive participants vs participants of unknown serostatus on their negative attitudes toward persons with HIV, exposure to normative attitudes, and their perceptions of normative attitudes, we could simply conduct three *t*-tests of the omnibus measures.

We used the omnibus measures in a multivariable regression analysis, following the procedures described by Imai and colleagues [95] and Imai and colleagues [96], to estimate the association between normative attitudes about persons with HIV (measured at the community level) and personal attitudes toward persons with HIV (measured at the individual level), and to determine the extent to which perceptions of normative attitudes (perceived stigma, also measured at the individual level) mediated this association. The parametric algorithm described in Imai and colleagues [95] computes two quantities of interest: the average causal mediation effect, which is the average change in personal attitudes toward persons with HIV corresponding to a change in perceived stigma under less vs more exposure to normative attitudes about persons with HIV; and the average direct effect, which is the average of all other causal mechanisms linking normative attitudes to personal attitudes. All of the omnibus measures were on the continuous scale, so we used standard linear regression models. The linear regression models adjusted for several socio-demographic and behavioral covariates as potential confounders, including sex, age, educational attainment, marital status, self-reported HIV seropositivity, and household asset wealth [67,86,97]. Cluster-correlated robust estimates of variance were used to account for clustering of study participants within villages [98-100].

The mediation analysis is contingent on causal assumptions holding for the total effect. Importantly, the mediation analysis assumes sequential ignorability, which requires that: (a) exposure to normative attitudes is statistically independent of potential outcomes and potential mediators; and (b) given exposure to normative attitudes and confounders, perceived stigma is also ignorable. The sequential ignorability assumption is likely untenable. Even if we had conducted a two-step randomized design in which exposure to both normative attitudes *and* perceived stigma were randomly assigned, such a design would still be unable to establish sequential ignorability [101,102]. Therefore, we conducted a sensitivity analysis to investigate the extent to which our mediation findings were robust to violations of the sequential ignorability assumption. Imai and colleagues [96] showed that violations of the sequential ignorability assumption lead to correlation between the error terms of the mediator model and the outcome model; thus we can calculate how high this correlation needs to be in order for the average causal mediation effect to be equal to zero. Similarly, we can use the sensitivity analysis to show how strong the omitted confounder must be (in terms of explaining remaining variance in both the mediator and the outcome) in order for the average causal mediation effect to be equal to zero.

In order to explore unmeasured confounding of the exposure-outcome relationship, we used the sensitivity analysis described in Oster [103]. The procedure assumes a value for the maximum R-squared associated with the regression model and then calculates a value for the relative degree of selection on unobserved vs observed variables (referred to as the “delta”) that would generate a regression coefficient equal to zero. For example, a delta equal to one would suggest that selection on observed variables is as at least as important as selection on unobserved variables; alternatively, a delta equal to two would suggest that selection on unobserved variables would need to be twice as important as selection on observed variables to generate a regression coefficient equal to zero. We followed Oster [103] in selecting a maximum R-squared value of 1.3 multiplied by the R-squared value that we obtained in the multivariable regression model with all covariates included, as this is the level of robustness that would be consistent with findings from randomized controlled trials.

## RESULTS

From June 2014-May 2015, we approached 1942 eligible persons for participation in the survey. Of these, 1776 (92%) consented to participate and were successfully interviewed. The number of participants per village ranged from 151 to 273. Characteristics of the sample are provided in **Table 1**. There were slightly more women than men in the parish, with more than one-half of the population aged 35 years or younger. Most participants had completed no more than primary school. The prevalence of self-reported HIV seropositivity was 8.8 percent.

**Table 1:** Characteristics of the sample (N = 1776)

	N	Proportion
Women	978	0.55
HIV-positive	156	0.09
**Age (years):**		
18-25	479	0.27
26-35	449	0.26
36-45	310	0.18
46-55	248	0.14
>55	257	0.15
**Educational attainment:**		
None	269	0.15
Some primary (P1-P6)	512	0.29
Completed primary (P7-P8)	394	0.22
Greater than primary	598	0.34
**Marital status:**		
Married, cohabitating	1036	0.58
Separated, divorced, widowed	318	0.18
Single, never married	417	0.24
**Household asset wealth:**		
Poorest (1st quintile)	351	0.20
Poorer	351	0.20
Middle	346	0.20
Less poor	349	0.20
Least poor (5th quintile)	332	0.19
**Village of residence:**		
Buhingo	273	0.15
Bushenyi	254	0.14
Nyamikanja I	232	0.13
Bukuna II	217	0.12
Nyakabare	151	0.09
Bukuna I	259	0.15
Rwembogo	150	0.08
Nyamikanja II	237	0.13

Among participants who reported their serostatus as HIV-negative or unknown, the prevalence of negative attitudes toward persons with HIV ranged from 2-52 percent, depending on the specific item (**Table 2**). In general, fewer participants endorsed the social distancing items compared with the items about social and economic worth. A smaller proportion of HIV-positive study participants, compared with participants who were HIV negative or of unknown serostatus, endorsed the items. On several items, very few to no HIV-positive participants endorsed the item; for example, no HIV-positive participants reported that they were unwilling to care for an HIV-positive relative, while only two HIV-positive participants reported that they were unwilling to buy food from an HIV-positive shopkeeper. In terms of perceptions of normative attitudes (perceived stigma), 23-46 percent of participants believed that most other people in their villages harbored these beliefs. On most, but not all, items, a smaller proportion of HIV-positive study participants, compared with participants who were HIV negative or of unknown serostatus, endorsed the item. Because there were no survey items on which the prevalence of agreement exceeded 50 percent, the data on perceptions of normative attitudes strongly suggest that a substantial percentage of study participants overestimated the extent to which negative attitudes toward persons with HIV were actually normative in their villages (**Table 3**).

**Table 2:** Proportion of study participants endorsing specific beliefs, stratified by self-reported serostatus (N = 1776)

	HIV-negative or unknown	HIV-positive
Unwilling to care for HIV-positive relative	0.02	0.00
Unwilling to buy food from HIV-positive shopkeeper	0.12	0.01
Would not permit HIV-positive teacher to teach children	0.14	0.03
Unwilling to share utensils with person with HIV	0.22	0.04
Would not permit person with HIV to prepare food for children	0.26	0.09
HIV is divine punishment	0.45	0.44
Persons with HIV have themselves to blame	0.52	0.28
Persons with HIV bring shame upon their family	0.35	0.20
Persons with HIV have reason to feel guilty	0.33	0.13
Persons with HIV not capable of providing food	0.13	0.04
Persons with HIV not capable of generating income	0.15	0.06
Persons with HIV are weaker, even if treated	0.44	0.29
Persons with HIV should not have children	0.36	0.21
Persons with HIV are of lower worth	0.25	0.14

**Table 3:** Perceived norms, stratified by self-reported serostatus (N = 1776)

	HIV-negative or unknown	HIV-positive
Most others unwilling to care for HIV-positive relative	0.45	0.40
Most others unwilling to buy food from HIV+ shopkeeper	0.26	0.17
Most others would not permit HIV-positive teacher to teach children	0.26	0.16
Most others unwilling to share utensils with person with HIV	0.39	0.31
Most others would not permit person with HIV to prepare food for children	0.44	0.34
Most others believe HIV is divine punishment	0.41	0.44
Most others believe persons with HIV have themselves to blame	0.46	0.42
Most others believe persons with HIV bring shame upon their family	0.31	0.29
Most others believe persons with HIV have reason to feel guilty	0.28	0.25
Most others believe persons with HIV not capable of providing food	0.23	0.17
Most others believe persons with HIV not capable of generating income	0.23	0.17
Most others believe persons with HIV are weaker, even if treated	0.42	0.42
Most others believe persons with HIV should not have children	0.37	0.38
Most others believe persons with HIV are of lower worth	0.24	0.28
Most others believe persons with HIV less able to contribute to community	0.23	0.23

*The items in this table parallel those described in **Table 2****.** The cell values refer to the proportion of study participants who believed that more than one-half of *other people* in the participant’s village would endorse the item in question.

In the exploratory factor analysis, a single dominant factor emerged with an eigenvalue of 3.64 that explained 73% of the variance. All of the items had substantial loadings on this factor, and the overall scale had a Cronbach’s alpha of 0.8. In addition, oblique promax rotation yielded a three-factor structure solution with a pattern of factor loadings that was easiest to interpret and consistent with prior theory. The first factor was most appropriately described as a “social distance” subscale and consisted of four items: willingness to purchase food from an HIV-positive shopkeeper, willingness to permit an HIV-positive teacher to teach, willingness to share dishes or utensils, and willingness to permit an HIV-positive person to prepare meals. The first factor had a Cronbach’s alpha of 0.8. The second factor was most appropriately described as a “blaming attitudes” subscale that consisted of four items: belief that HIV is divine punishment, assigning blame to HIV-positive persons, belief that HIV brings shame, and assigning guilt to HIV-positive persons. The second factor had a Cronbach’s alpha of 0.7. The third factor was most appropriately described as a “reciprocity concerns” subscale that consisted of four items: belief that HIV-positive persons are equally capable of providing food, belief that HIV-positive persons are equally capable of generating income, belief that HIV-positive persons are of lower worth, and belief that HIV-positive persons are less able to contribute to the community. The third factor had a Cronbach’s alpha of 0.7. Three items that were included in the dominant factor did not load heavily on any of these three subscales: willingness to care for an HIV-positive relative, belief that HIV-positive persons are physically weaker, and belief that HIV-positive persons should not have children. In the remainder of this analysis, we proceeded with the single omnibus measure.

The omnibus tests were consistent with the patterns observed on the individual scale items. Compared with HIV-positive study participants, participants who were HIV negative or of unknown serostatus harbored a greater intensity of negative attitudes toward persons with HIV on the omnibus measure (0.03 vs -0.28; *t* = 7.2, *P* < 0.001) (**Table 2**). A similar pattern was observed for anticipated stigma, with participants who were HIV negative or of unknown serostatus reporting that they perceived normative attitudes to be more stigmatizing than did HIV-positive participants (0.01 vs -0.12; *t* = 3.0, *P* = 0.002) (**Table 3**).

Between villages, negative attitudes toward persons with HIV varied by as much as a factor of two to three, depending on the specific item, with coefficients of variation ranging from 13-48 percent (**Table 4**). Comparing the prevalence of agreement on the survey items to the perceptions of the normative attitudes, we found that 22-70 percent of participants, depending on the specific item, overestimated the extent to which others in their villages actually harbored negative attitudes toward persons with HIV. Between 0-45 percent of participants made underestimates. For most of the items, the percentage of participants who overestimated the extent of perceived stigma exceeded the percentage of participants who underestimated the extent of perceived stigma. On the omnibus measure, participants who were HIV negative or of unknown serostatus demonstrated a greater tendency to overestimate the extent of anticipated stigma, compared with HIV-positive participants (0.01 vs -0.09; *t* = 2.4, *P* = 0.02). In contrast, persons with HIV demonstrated a greater tendency to underestimate the extent of anticipated stigma, compared with HIV-negative participants or participants of unknown serostatus (0.12 vs -0.01; *t* = 3.1, *P* = 0.002).

**Table 4:** Variation in negative attitudes toward persons with HIV, across villages (N = 8)

	Mean pct.	SD	Min pct.	Max pct.	CV
Pct. unwilling to care for HIV-positive relative	2.16	1.03	0	3.33	0.48
Pct. unwilling to buy food from HIV-positive shopkeeper	10.79	2.32	6.95	14.00	0.21
Pct. who would not permit HIV-positive teacher to teach children	12.74	3.11	7.75	16.88	0.24
Pct. unwilling to share utensils with person with HIV	21.30	4.19	13.90	28.67	0.20
Pct. who would not permit person with HIV to prepare food for children	24.83	3.13	18.92	29.03	0.13
Pct. who believe HIV is divine punishment	44.72	5.79	36.36	55.70	0.13
Pct. who believe persons with HIV have themselves to blame	50.38	6.72	40.93	60.00	0.13
Pct. who believe persons with HIV bring shame upon their family	34.02	7.18	23.55	42.00	0.21
Pct. who believe persons with HIV have reason to feel guilty	31.79	7.19	19.31	41.35	0.23
Pct. who believe persons with HIV not capable of providing food	12.38	2.64	9.48	17.72	0.21
Pct. who believe persons with HIV not capable of generating income	14.34	1.84	12.36	17.05	0.13
Pct. who believe persons with HIV are weaker, even if treated	42.78	7.77	33.82	51.90	0.18
Pct. who believe persons with HIV should not have children	34.71	5.04	29.15	43.46	0.15
Pct. who believe persons with HIV are of lower worth	24.22	5.57	16.60	33.76	0.23
Pct. who believe persons with HIV less able to contribute to community	19.65	4.88	12.36	27.43	0.25

CV – coefficient of variation, SD – standard deviation, Pct – percentage

* The items in this table parallel those described in **Table 2****.** The cell values in the “Mean pct.” column refer to the mean percentage of study participants, across villages, who endorsed the item in question. The cell values in the “Min pct.” column refer to the village with the smallest percentage of study participants who endorsed the item. The cell values in the “Max pct.” column refer to the village with the largest percentage of study participants who endorsed the item.

In our multivariable regression analysis, we confirmed that: (1) participants who lived in villages with a greater intensity of negative attitudes toward persons with HIV were more likely themselves to hold negative attitudes toward persons with HIV (b = 0.10; 95% confidence interval (CI) = 0.08-0.12); (2) participants who lived in villages with a greater intensity of negative attitudes toward persons with HIV were more likely to perceive these negative attitudes as normative (b = 0.08; 95% CI = 0.04-0.11); and (3) the association between normative attitudes and personal attitudes was substantially reduced in magnitude and statistical significance (b = 0.06; 95% CI = 0.03-0.09) after adjusting for perceived norms. In total, 40.7 percent of the total association between normative attitudes and personal attitudes was mediated by perceived norms (perceived stigma).

In the sensitivity analysis, we found that in order for the average causal mediation effect to be zero, the correlation between the error terms of the mediator model and the outcome model would need to be quite high (ρ = 0.56). Alternatively stated, the product of the R-squared values for both models would have to be 0.31 in order for the average causal mediation effect to be zero. Such a product would be equivalent to, for example, an omitted confounder explaining 60% of remaining variance in perceived stigma and 50% of remaining variance in personal attitudes (0.6 × 0.5 = 0.3). In exploring the robustness of the exposure-outcome relation, the R-squared from the regression model with all covariates was 0.37, so we assumed a maximum R-squared value of 0.37 × 1.3 = 0.481 in applying the procedures described by Oster [103]. We calculated a delta of 3.6 (standard error = 0.67), indicating that the model for the association between normative attitudes and personal attitudes is fairly robust: selection on unobserved variables would need to be three times as important as selection on observed variables to generate a regression coefficient equal to zero.

## DISCUSSION

In this cross-sectional, population-based survey from rural Uganda, we found that HIV remains heavily stigmatized despite the introduction and scale-up of ART [10,13,72,104,105]. Our findings provide strong support for two of our three study hypotheses: study participants believed that negative attitudes toward persons with HIV were more pervasive than they actually are, and their perceptions of the extent to which these negative attitudes are normative mediated the association between normative attitudes and their own personal attitudes. The evidence directly contradicted our third hypothesis, as persons with HIV were actually *less* likely to misperceive these norms compared with participants of unknown serostatus. Our findings have important implications for research and intervention programs, which we describe in detail below.

Our finding that study participants frequently overestimated the pervasiveness of negative attitudes toward persons with HIV is consistent with previously published findings about how young adults in high-income countries frequently overestimate the pervasiveness of hazardous alcohol use [35-39]. Stated differently, study participants believed that other persons in their villages had much more stigmatizing beliefs than they actually did. These documented misperceptions could be consistent with the phenomenon of pluralistic ignorance [106], ie, most of our study participants falsely assumed that most others in the village harbored more negative attitudes toward persons with HIV when in fact their attitudes were more similar than they thought. Our study was not designed to identify the drivers of these misperceptions. For example, it is possible that study participants who observed enactments of stigma [63] – or even heard about such enactments, through word of mouth – may have assigned disproportionate salience to these events (ie, due to the consequences of social rejection or physical harm) and that this salience could have led them to overestimate the extent to which negative attitudes toward persons with HIV are actually normative.

In partial support of our hypothesis, we found that perceptions about the extent to which negative attitudes toward persons with HIV are normative (perceived stigma) partially mediated the association between normative attitudes and personal attitudes. Theorists in social psychology have long held that individual perceptions and behavior may be influenced by observations and perceptions of the behavior of others [107]. For example, Festinger [108] theorized that people use social comparison processes to evaluate their own beliefs relative to social reality. More recently, in an analysis of the stigma of mental illness, Link and colleagues [44] proposed a labeling model in which societal conceptions of what it means to be living with mental illness were first perceived by persons with mental illness, and then adopted as relevant, thereby leading to adverse consequences for health and well-being. In our data, more than one-third of the total association between normative attitudes and personal attitudes was mediated by perceived stigma. Our sensitivity analyses showed that confounding (either of the mediator-outcome relation or of the exposure-outcome relation) by an unobserved variable would need to be quite strong in order for the total and average causal mediation effects to be zero, thereby suggesting the robustness of this finding.

A secondary contribution of this study is the introduction of a validated scale for measuring negative attitudes toward persons with HIV that can also be used to measure perceived stigma in a general population sample or anticipated stigma among persons with HIV. The 15-item scale demonstrated a coherent factor structure that was internally consistent. Further analysis revealed a three-factor solution with three related, but distinct, constructs: social distancing, blaming attitudes, and reciprocity concerns. The three subscales were internally consistent. Previously published studies conducted in a variety of settings have elaborated scales to measure social distancing [68,83] and blaming attitudes [66,84,85]. However, we also introduce a new subscale to measure instrumentally- and symbolically-driven concerns about the ability of persons with HIV to make reciprocal economic contributions to local networks of mutual aid. This aspect of HIV stigma has been described in qualitative and ethnographic studies conducted in multiple settings throughout sub-Saharan Africa [59-61,109-113], thereby suggesting strong construct validity. However, this construct has not been measured in studies of population health. Our subscale is perhaps closest in description to Maughan-Brown’s [85] conceptualization of “resource-based stigma” in South Africa, which he defines as negative attitudes toward persons with HIV driven by instrumental resentment of the resources expended on their support, eg, through the governmental disability grant [114].

### Limitations

Interpretation of our findings is subject to several important limitations. First, our measure of normative attitudes could have been measured with error. As explicated by Borsari and Carey [38] and Lapinski and Rimal [115], collective norms exist at multiple social levels. Therefore, measuring norms at the village level may have provided inadequate resolution to understand social influences on perceptions and beliefs. Studies that measure norms at the level of the social network [116,117] may provide better traction to investigate these phenomena in more detail. Second, our findings could have been affected by social desirability bias [118]. HIV prevention campaigns have been widely disseminated throughout Uganda [119,120], and they could have sensitized participants to knowing what they *ought* to believe about persons with HIV without actually changing what they actually believe. This bias likely would have shifted our estimated associations (between normative attitudes, personal attitudes, and perceptions of the norm) away from the null. However, our sensitivity analyses suggest that selection bias would need to be quite strong in order to undermine our findings. Third, HIV serostatus with HIV testing was self-reported. While it is rare for persons to self-report HIV seropositivity when they are actually seronegative [89], it is possible that some persons self-reported HIV seronegativity when they were in fact seropositive. However, our estimate of self-reported HIV seropositivity closely matched the HIV prevalence estimate for southwestern Uganda in the 2011 Uganda AIDS Indicator Survey that was based on unlinked anonymous HIV testing [121]; therefore we anticipate that any potential bias resulting from misclassification would be minimal.

## CONCLUSIONS

In a rural area of Uganda where HIV stigma has persisted after a decade of intensive ART scale-up activity, we found evidence of pervasive misperceptions about the extent to which negative attitudes toward persons with HIV are normative. We further found that these misperceptions mediated the association between normative attitudes and personal attitudes. An important implication of our findings is that interventions designed to accurately convey norms (ie, by informing recipients what most other people in their communities believe) may reduce HIV stigma without directly focusing on the educational components that are typically embedded in anti-stigma interventions (ie, by informing recipients what they themselves *should* believe) [122]. In the context of drinking among young adults in high-income countries, interventions targeting misperceived norms have reduced hazardous alcohol consumption and misperceptions of hazardous alcohol consumption [123]. As an example of related work from sub-Saharan Africa, Dupas [124] conducted a field experiment in which Kenyan adolescent girls were provided with accurate information about the age distribution of HIV prevalence among men, thereby leading to selection of younger-age partners and reductions in teenage pregnancy. Notably, it is possible that such interventions could have unintended consequences: for example, conveying accurate information about negative attitudes toward persons with HIV could have a “boomerang” effect by intensifying negative attitudes among those who do not already hold them [125]. We believe these types of interventions warrant further testing so that the stigma of HIV can be effectively addressed worldwide.

## Additional material


Online Supplementary Document
