# Supplementary material for: Normative vs personal attitudes toward persons with HIV, and the mediating role of perceived HIV stigma in rural Uganda
Source: J Glob Health. 2021 Sep 15;11:04956. doi: 10.7189/jogh.11.04056 (PMC8442577; doi:10.7189/jogh.11.04056)
Supplement: Online Supplementary Document [file jogh-11-04056-s001.pdf]

Table S1. Survey questions used to elicit personal beliefs and perceived norms about HIV

Personal beliefs

In your personal opinion, would you be willing to...

1. ...care for a relative who is sick in bed because of HIV/AIDS?
2. ...buy food from a shopkeeper you know is HIV-positive?
3. ...permit an HIV-positive teacher to teach your own children or the children of your family members?
4. ...share the same dishes or utensils, or the same cup, with someone you know is HIV-positive?
5. ...permit an HIV-positive person to prepare meals for your own children or the children of your family members?

In your personal opinion, do you agree or disagree with the following statement?

6. HIV/AIDS is a divine punishment for doing immoral things.
7. People who get infected with HIV through sexual intercourse have only themselves to blame.
8. HIV brings shame upon one's entire family.
9. HIV-positive persons have reason to feel guilty for being infected with HIV.
10. HIV-positive persons should be regarded as equally capable as HIV-negative persons in terms of providing food for the household.
11. HIV-positive persons should be regarded as equally capable as HIV-negative persons in terms of generating income for the household.
12. Even if they receive antiretroviral treatment, HIV-positive persons are physically weaker than HIV-negative persons.
13. HIV-positive persons should not have children.
14. HIV-positive persons are of lower worth compared to HIV-negative persons.
15. HIV-positive persons are less able to contribute to the community compared to HIV-negative persons.

Perceived norms

According to your perception, how many other people aged 18 years or older who stay in your cell, not including yourself, would be willing to...

16. ... care for a relative who is sick in bed because of HIV/AIDS?
17. … buy food from a shopkeeper they know is HIV-positive?

18. … permit an HIV-positive teacher to teach people's own children or the children of their family members?
19. … share the same dishes or utensils, or the same cup, with someone they know is HIV-positive?
20. … permit an HIV-positive person to prepare meals for people's own children or the children of their family members?

According to your perception, how many other people aged 18 years or older who stay in your cell, not including yourself, would agree with the following statement?

21. HIV/AIDS is a divine punishment for doing immoral things.
22. People who get infected with HIV through sexual intercourse have only themselves to blame.
23. HIV brings shame upon one's entire family.
24. HIV-positive persons have reason to feel guilty for being infected with HIV.
25. HIV-positive persons should be regarded as equally capable as HIV-negative persons in terms of providing food for the household.
26. HIV-positive persons should be regarded as equally capable as HIV-negative persons in terms of generating income for the household.
27. Even if they receive antiretroviral treatment, HIV-positive persons are physically weaker than HIV-negative persons.
28. HIV-positive persons should not have children.
29. HIV-positive persons are of lower worth compared to HIV-negative persons.
30. HIV-positive persons are less able to contribute to the community compared to HIV-negative persons.
